# Draft genome sequences of two bacterial type strains isolated from a methanogenic bioreactor: *Ruminiclostridium sufflavum* DSM 19573^T^ and *Anaerosphaera aminiphila* DSM 21120^T^

**DOI:** 10.1128/mra.00578-25

**Published:** 2025-07-31

**Authors:** James Cusack, Itzel Del Rio, Stefan Spring, Nikos Kyrpides, Natalia Ivanova, Rekha Seshadri, Matthew Escobar

**Affiliations:** 1 Department of Biological Sciences, California State University San Marcos 548796, San Marcos, California, USA; 2 Leibniz Institute DSMZ, German Collection of Microorganisms and Cell Cultures, Braunschweig, Germany; 3 Department of Energy Joint Genome Institute, Lawrence Berkeley National Laboratory 1666, Berkeley, California, USA; University of Maryland School of Medicine, Baltimore, Maryland, USA

**Keywords:** *Ruminiclostridium sufflavum*, *Anaerosphaera aminiphila*, draft genome sequence, bioreactors, amino acid fermentation

## Abstract

*Ruminiclostridium sufflavum* DSM 19573^T^ and *Anaerosphaera aminiphila* DSM 21120^T^ were first isolated from a methanogenic bioreactor treating cattle waste in Hokkaido, Japan. The *R. sufflavum* draft genome sequence is 4.4 Mb with 3,773 predicted genes, and the *A. aminiphila* draft genome sequence is 2.0 Mb with 1,962 predicted genes.

## ANNOUNCEMENT

Anaerobic digestion depends on complex microbial communities that convert organic waste into biogas and nutrient-rich fertilizer. Methane-producing anaerobic bioreactors are commonly used to process livestock waste, and microorganisms in these bioreactors must efficiently ferment lipids, carbohydrates, and proteins (1). *Ruminiclostridium sufflavum* DSM 19573^T^ (CDT-1^T^) and *Anaerosphaera aminiphila* DSM 21120^T^ (WN036^T^) were isolated from rice straw residue in a methanogenic bioreactor used to treat cattle waste in Hokkaido, Japan (2–5). *R. sufflavum* DSM 19573^T^ is an anaerobic Gram stain-variable bacillus that is motile via peritrichous flagella. It ferments the polysaccharides cellulose and xylan, with acetate and ethanol as primary products (3). *A. aminiphila* DSM 21120^T^ is an anaerobic, nonmotile, Gram stain-variable coccus. It primarily ferments amino acids, producing acetate and butyrate as major products (2, 5). The draft genome sequences of these bacterial type strains will expand our understanding of amino acid and carbohydrate fermentation in waste treatment bioreactors.

*R. sufflavum* DSM 19573^T^ and *A. aminiphila* DSM 21120^T^ were sequenced by the Joint Genome Institute’s 1000 Microbial Genomes Project in 2014 (6). Freeze-dried cultures from DSMZ were used to inoculate 250 mL of DSMZ 110 medium (*R. sufflavum*) or 1500 mL of DSMZ 104b medium (*A. aminiphila*), with growth at 30°C for one day. Anaerobic conditions (100% N_2_) were established in sealed serum vials using the Hungate technique (7, 8). Genomic DNA was isolated from culture cell pellets using an Invitrogen Jetflex Genomic DNA Purification Kit (*R. sufflavum*) or a Lucigen MasterPure gram-positive DNA Purification Kit (*A. aminiphila*). DNA was sheared to ~300 bp using a Covaris LE220 ultrasonicator. Library preparation was performed using a KAPA Biosystems library preparation kit with Illumina-compatible adaptors. An Illumina TruSeq paired-end cluster kit (v.4) was then used to generate a clustered flow cell. A 2 × 150 indexed sequencing run was performed on an Illumina HiSeq 2000 using Illumina HiSeq TruSeq SBS sequencing kits (v.3 for *A. aminiphila*, v.4 for *R. sufflavum*). Read filtering was performed using DUK (v.1.0) (9); genome assembly was performed using Velvet (v.1.2.07), wgsim (v.1.0), and Allpaths-LG (v.r46652) (10–12); and genome annotation was performed using Prodigal (v.2.5) and GenePRIMP (v.1.0) (13, 14), with all software settings as previously described (15). An overview of the genomes is provided in Table 1.

**TABLE 1:** Genomic features of *Ruminiclostridium sufflavum* DSM 19573^T^ and *Anaerosphaera aminiphila* DSM 21120^T^

Genome feature	*R. sufflavum* DSM 19573^T^	*A. aminiphila* DSM 21120^T^
Total number of sequencing reads	7,329,322	6,439,906
Total DNA sequenced (Mbp)	1,099.4	966.0
Assembled draft genome size (bp)	4,401,411	2,021,907
Number of scaffolds	57	32
Scaffold N_50_ (bp)	172,915	122,354
Average fold coverage	248×	467×
CheckM2 genome completeness (16)	100%	99.6%
Check M2 genome contamination (16)	0.16%	0.04%
GC content	38.62%	31.54%
Predicted genes	3,773	1,962
Predicted protein-coding genes	3683	1916
Predicted rRNAs	9	9
Predicted tRNAs	52	32
Joint Genome Institute IMG/M taxon ID	2599185205	2585428066
NCBI WGS accession number	GCF_003208175.1	GCA_900129925.1
NCBI Bioproject accession number	PRJNA262320	PRJNA245635
NCBI Sequence Read Archive accession number	SRR4140139	SRR4096543
NCBI BioSample number	SAMN05660363	SAMN02745245

**Fig 1:**
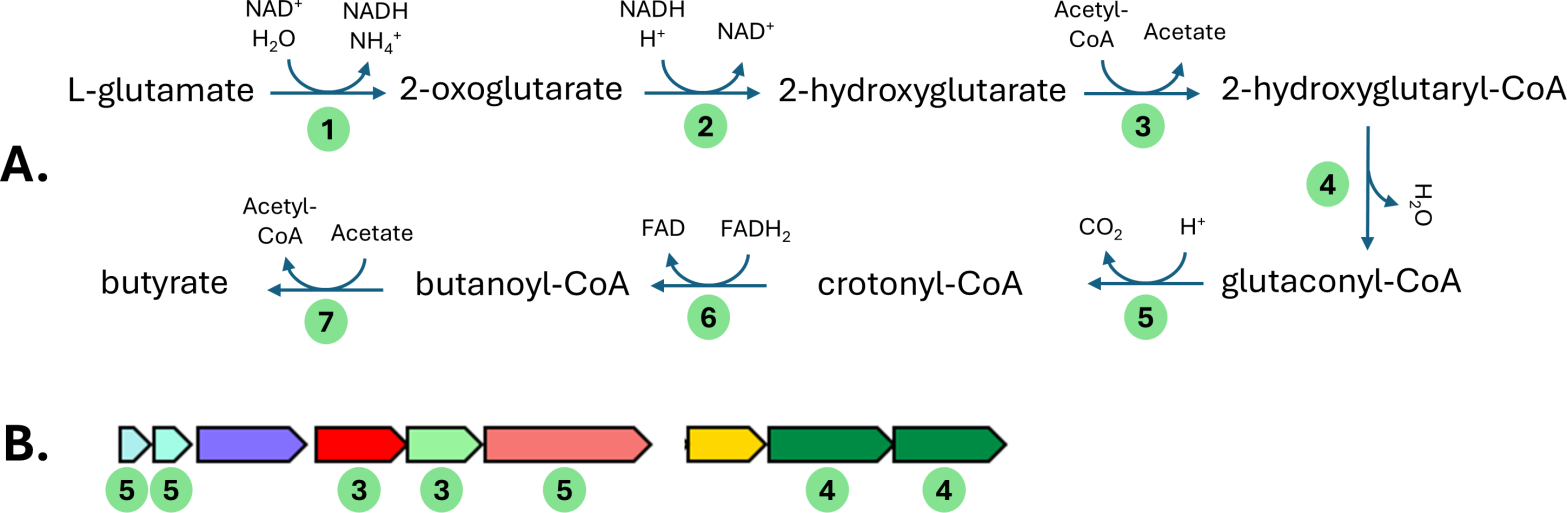
Putative 2-hydroxyglutarate-based glutamate fermentation pathway in *A. aminiphila* DSM 21120^T^. (**A**) 2-hydroxyglutarate pathway, with green circles indicating the presence of putative genes encoding pathway enzymes. 1. Glutamate dehydrogenase, Joint Genome Institute (JGI) Gene ID 2587768148; 2. 2-oxoglutarate reductase, JGI Gene IDs 2587768177 and 2587768289; 3. glutaconate CoA-transferase, JGI Gene IDs 2587769252 (subunit A) and 2587769253 (subunit B); 4. 2-hydroxyglutaryl-CoA dehydratase, JGI Gene IDs 2587769256 (subunit alpha) and 2587769257 (subunit beta); 5. glutaconyl-CoA decarboxylase, JGI Gene IDs 2587769254 (subunit alpha), 2587769249 (subunit delta), and 2587769250 (subunit gamma); 6. butanoyl-CoA dehydrogenase, JGI Gene ID 2587768393; 7. butanoyl-CoA transferase. JGI Gene ID 2587768328. (**B**) A series of consecutive *A. aminiphila* DSM 21120^T^ operons containing genes encoding putative glutamate fermentation pathway enzymes, as indicated.

Amino acid fermentation in bacteria has been poorly characterized compared to carbohydrate fermentation (17–19). *R. sufflavum* DSM 19573^T^ ferments cellulose and xylan (3), and genome analyses using IMG/M (20) revealed 22 putative cellulases (genes with glycoside hydrolase 5/8/9/44/48 domains) and three putative xylanases (genes with glycoside hydrolase 10/11 domains). *A. aminiphila* DSM 21120^T^ ferments amino acids, including glutamate (2). Two major glutamate fermentation pathways have been described in bacteria: the 2-hydroxyglutarate pathway and the 3-methylaspartate pathway (21). While the *A. aminiphila* DSM 21120^T^ genome lacks genes encoding the enzymes in the 3-methylaspartate pathway, genes encoding all 2-hydroxyglutarate pathway enzymes appear to be present, many organized in a set of consecutive operons (Fig. 1). Further research is needed to verify the operation of the 2-hydroxyglutarate pathway in *A. aminiphila* DSM 21120^T^.
